# Fgf8-Deficient Mice Compensate for Reduced GnRH Neuronal Population and Exhibit Normal Testicular Function

**DOI:** 10.3389/fendo.2015.00151

**Published:** 2015-09-22

**Authors:** Wei Zhang, Joshua I. Johnson, Pei-San Tsai

**Affiliations:** ^1^Department of Integrative Physiology, Center for Neuroscience, University of Colorado Boulder, Boulder, CO, USA

**Keywords:** GnRH, *Fgf8*, hypogonadotropic hypogonadism, development, puberty, testes

## Abstract

Gonadotropin-releasing hormone (GnRH) is critical for the onset and maintenance of reproduction in vertebrates. The development of GnRH neurons is highly dependent on fibroblast growth factor (Fgf) signaling. Mice with a hypomorphic *Fgf8* allele (*Fgf8* Het) exhibited a ~50% reduction in GnRH neuron number at birth. Female *Fgf8* Het mice were fertile but showed significantly delayed puberty. However, it was unclear if these mice suffered additional loss of GnRH neurons after birth, and if male *Fgf8* Het mice had normal pubertal transition and testicular function. In this study, we examined postnatal GnRH neuron number and hypothalamic GnRH content in *Fgf8* Het mice from birth to 120 days of age. Further, we examined seminal vesicle and testicular growth, testicular histology, and circulating luteinizing hormone (LH) around and after pubertal transition. Our results showed that GnRH neuron numbers were significantly and consistently reduced in *Fgf8* Het mice of both sexes in all ages examined, suggesting these animals were born with an inherently defective GnRH system, and no further postnatal loss of GnRH neurons had occurred. Despite an innately compromised GnRH system, male and female *Fgf8* mice exhibited normal levels of immunoassayable hypothalamic GnRH peptide at all ages examined except on 60 days of age, suggesting increased GnRH synthesis or reduced turnover as a compensatory mechanism. *Fgf8* Het males also had normal seminal vesicle and testicular mass/body mass ratios, testicular histology, and circulating LH. Overall, our data speak to the extraordinary ability of a GnRH system permanently compromised by developmental defect to overcome pre-existing deficiencies to ensure pubertal progression and reproduction.

## Introduction

Neurons that synthesize and secrete gonadotropin-releasing hormone (GnRH) are critical to vertebrate reproduction. The functional network of GnRH neurons develops via a multi-step process that includes GnRH neuronal fate specification in the nose, migration to the forebrain, and axon targeting to the median eminence. GnRH released from the mature GnRH system is ultimately responsible for the stimulation of pituitary gonadotropin secretion, and hence gonadal maturation and reproduction. The development of GnRH neurons is orchestrated by a multitude of signaling factors (1–3). The importance of these factors is underscored by observations that loss-of-function mutations in many of these signaling molecules could lead to hypogonadotropic hypogonadism (HH) in humans, a disorder characterized by low circulating gonadotropins and absent puberty.

Fibroblast growth factor (Fgf) signaling is a large family of genes that comprises 22 ligands, 4 receptors, several ligand and receptor splice variants, and genes synexpressed with Fgf ligands (4–13). Fgf signaling is indispensable for the development, survival, and maturation of the central nervous system (9, 13). Mounting evidence from human studies reveals loss-of-function mutations on *Fgf receptor (Fgfr) 1*, *Fgf8*, *Fgf17*, and synexpression genes such as *IL17RD*, *DUSP6*, *SPRY4*, and *FLRT3* are causal to HH (14–16). Studies in mice confirm that many of the Fgf signaling genes are expressed prominently in the olfactory placode, the birthplace of GnRH neurons (16, 17), and their deficiencies could lead to severely reduced GnRH neuronal population (15, 18–22). Of these, *Fgf8* is particularly critical for the development of GnRH neurons. Heterozygous *Fgf8* hypomorphic mice (*Fgf8* Het) exhibit a 50% reduction in GnRH neurons at birth (20), whereas newborn homozygous *Fgf8* hypomorphic mice exhibit a complete elimination of GnRH neurons due to fate specification failure (20).

Although the role of Fgf8 signaling in GnRH neuronal development has been reported, two questions remain unanswered. First, deficiency in *Fgfr3*, one of the cognate receptors for Fgf8 (23), results in the postnatal loss of GnRH neurons without affecting their development (20, 21). It is currently unclear if *Fgf8* also contributes to the postnatal maintenance of the GnRH system in addition to its early effects on the genesis of GnRH neurons. Second, an interesting paradox is that while a fraction of humans with *Fgf8* haploinsufficiency experiences absent puberty and infertility (15, 24), *Fgf8* Het mice consistently go through puberty and propagate offspring (25). This raises the possibility that the GnRH system of *Fgf8* Het mice may compensate for the loss of GnRH neurons by upregulating the production of GnRH. Interestingly, although *Fgf8* Het female mice are fertile, they exhibit delayed vaginal opening (VO) and first estrus (26). It is at present unclear if *Fgf8* Het male mice exhibit normal gonadal function during and after puberty.

The objectives of this study are twofold. First, we examine if *Fgf8* Het mice suffer an additional loss of GnRH neurons after birth, and if a smaller GnRH neuronal population resulting from *Fgf8* deficiency could produce normal levels of the GnRH peptide to offset the overall neuronal loss. Second, by measuring the growth and maturation of testes and seminal vesicle (SV) as well as circulating luteinizing hormone (LH) level, we examine if male *Fgf8* Het mice undergo normal pubertal progression compared to wildtype (WT) controls. Our results show that *Fgf8* deficiency obliterates half of GnRH neuronal population prenatally and permanently in both sexes, but all mice compensate for this innate GnRH system deficit by increasing the levels of the GnRH peptide before, during, and after puberty with only some rare exceptions. Further, male *Fgf8* Het mice have normal circulating LH and undergo testicular growth and spermatogenesis comparable to WT controls. Our results highlight the ability of an inherently defective murine GnRH system to overcome a reduction in its neuronal population in order to facilitate pubertal transition and support reproduction in adulthood.

## Materials and Methods

### Animals

*Fgf8* Het mice (129P2/OlaHsd* CD-1) (25) were initially obtained from the Mouse Regional Resource Centers (Davis, CA, USA) and maintained in the University of Colorado animal facility under a 12L:12D cycle and fed rodent chow and water *ad libitum*. *Fgf8* hypomorphic mice were generated by the insertion of a neo cassette in the non-coding region of the *Fgf8* gene, resulting in the abnormal splicing of *Fgf8* transcript. Homozygous *Fgf8* hypomorphs showed a 54% decrease in functional *Fgf8* message, died within 24 h of birth (25), and were not used in this study. *Fgf8* Het mice, however, were viable and propagated offspring. WT and *Fgf8* Het used in this study were derived from the breeding of *Fgf8* Het × *Fgf8* Het or WT × *Fgf8* Het. The day of birth was designated as postnatal day (PN) 0. Mice were weaned at approximately PN20 and housed separately by sex after weaning. All animals were genotyped by polymerase chain reaction of genomic DNA isolated from tail biopsies. All animal procedures complied with protocols approved by the Institutional Animal Care and Use Committee at the University of Colorado.

### GnRH immunohistochemistry and quantification of GnRH neurons

GnRH immunohistochemistry (IHC) was carried out as described previously (20, 21). Briefly, the brain region containing the preoptic area (POA) was dissected by blocking anteriorly at the caudal border of the olfactory bulbs and posteriorly at the optic chiasm. Blocked tissues were immersion-fixed in 5% acrolein in 0.1M phosphate buffer for 6 h (for PN0) or overnight (for older animals), and then cryoprotected in 30% sucrose. Fixed brain tissues were sectioned at 50-μm thickness in a cryostat and transferred into a cryoprotectant containing 30% sucrose, 1% polyvinylpyrolidone, and 30% ethylene glycol in 0.05M phosphate buffer, washed in 0.1M phosphate-buffered saline (PBS), and incubated with 1% sodium borohydride in PBS for 40 min to quench the activity of residual acrolein. Sections were then washed extensively in PBS, incubated with 1% H_2_O_2_ in PBS for 15 min to quench the endogenous peroxidase activity, and then washed in PBS containing 0.4% Triton X (PBST). All sections were incubated with a polyclonal antibody against GnRH (LR5; from Dr. Robert Benoit, McGill University Health Center, Montréal, QC, Canada; 1:20,000) diluted in PBST containing 4% normal donkey serum and 10% normal horse serum for 5 days at 4°C. After 5 days, sections were washed in PBST, incubated with a biotinylated donkey anti-rabbit IgG (1:500) for 1.5 h, washed in PBST, incubated with an avidin-biotin-peroxidase complex (Vector Labs, Burlingame, CA, USA) for 1 h, washed in PBST, and visualized using diaminobenzidine as a chromagen. Sections were then mounted onto gelatin-coated slides, dehydrated through graded ethanol series and coverslipped. GnRH neurons within set distances anterior (1500 μm for PN0–10, 2100 μm for PN20–35, 2450 μm for PN60, 2700 μm for PN120) and posterior (500 μm for PN0, 650 μm for PN10, 700 μm for PN20–35, 900 μm for PN60, 1100 μm for PN120) to the organum vasculosum of the lamina terminalis (OVLT) were scored for GnRH neurons by an investigator blind to the identity of the slides. Although these set distances may exclude a few of the most anterior and posterior GnRH neurons, this method ensured that the number of sections quantified was consistent among all animals within the same age group. Only positively stained cells that appeared neuronal and exhibited clear nuclei were scored.

### GnRH radioimmunoassay

Hypothalami were removed by blocking anteriorly at the optic chiasm, posteriorly at the anterior border of the mammillary body, sagittally by two cuts along the lateral borders of the hypothalamus, and dorsally with one cut to remove the cortex. Tissues were immediately flash-frozen on pulverized dry ice and stored at −70°C until further processing. For GnRH extraction, tissues were sonicated in 0.1M phosphate buffer on ice, boiled for 2 min, flash-frozen on dry ice and stored at −70°C until GnRH radioimmunoassay (RIA). Gonadotropin-releasing hormone RIA was carried out using a GnRH-specific antiserum (R1245, provided by T. M. Nett at Colorado State University). Detailed protocol for GnRH RIA was described elsewhere (27). The detection limit was 1.87 pg/tube. The intra- and inter-assay coefficients of variation were 7.3 and 5.0%, respectively.

### LH radioimmunoassay

Trunk blood from decapitated PN35 and PN60 male WT and *Fgf8* Het mice was collected into heparinized tubes and the plasma isolated by centrifugation. Plasma LH was measured using a rat LH RIA as previously described (28). The detection limit was 0.06 ng/ml. The intra- and inter-assay coefficients of variation were 9.3 and 10.1%, respectively.

### Seminal vesicle and testicular parameters

PN20, 35, 40, and 60 WT and *Fgf8* Het male mice were weighed before sacrifice. Testes and SV from all animals were dissected and weighed, and all testes were immersion-fixed in Bouin’s fixative overnight. Fixed testes were stored in 70% ethanol, dehydrated in increasing concentrations of ethanol, cleared in Histoclear (National Diagnostics, Atlanta GA, USA), and embedded in paraffin. Serial sections were cut at 12-μm coronally on a rotary microtome, mounted on slides and stained with hematoxylin and eosin before dehydration and coverslipping.

### Morphometric analysis

Eight testicular sections, each five sections from the next, were scored for each animal. For each section, five seminiferous tubules (STs) were randomly selected from each of the following five zones: upper, lower, left, right, and middle portions of the testicular cross-section. Therefore, a total of 200 STs were measured for each testis. Total and luminal diameters of each ST were measured by a calibrated ocular micrometer. Since most STs were elliptical in shape, each total or luminal diameter measurement consisted of an average of two scores: one taken at the widest and one at the narrowest part. The number of ST with open lumen and mature sperm in each scored section was also recorded. Only clear opening with rounded boundary were considered as an open lumen. Mature sperm was identified by the appearance of both the head and the tail in the lumen.

### Statistical analysis

Differences in GnRH neuron number, hypothalamic GnRH, plasma LH, testicular/body mass and SV/body mass ratios, and histological measurements between genotypes and among ages were analyzed by two-way ANOVA followed by Bonferroni’s *post hoc* test. Differences were considered significant when *P* < 0.05. To analyze the percentage of GnRH neurons lost after birth, GnRH neurons number for each *Fgf8* Het mouse was first normalized to the mean of GnRH neuron numbers in WT mice of same sex and age. The normalized numbers (% control GnRH neurons) were analyzed by Kruskal–Wallis test followed by Dunn’s *post hoc* test.

## Results

**Figure 1:**
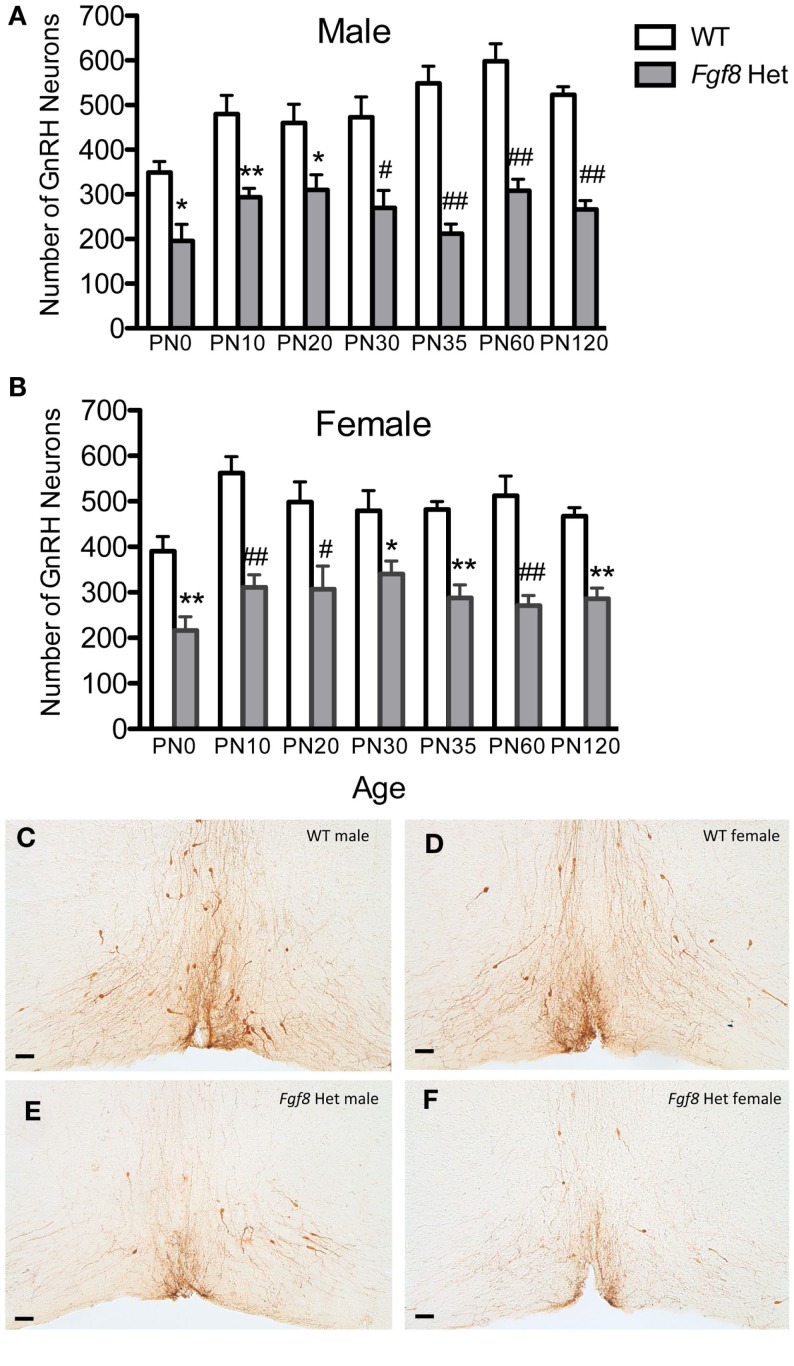
**Number of GnRH neurons in PN0–120 male (A) and female (B) WT and *Fgf8* Het mice**. In both sexes, GnRH neuron numbers in *Fgf8* Het mice were consistently reduced compared to WT at all ages examined. Each bar = mean ± SEM; *N* = 5–6. *P < 0.05; **P < 0.01; ^#^*P* < 0.001; ^##^*P* < 0.0001 compared to WT of the same age. **(C–F)** Representative photomicrographs of GnRH IHC at the plane of OVLT in PN35 WT **(C,D)** and *Fgf8* Het **(E,F)** male and female mice. Scale bar = 50 μm.

**Figure 2:**
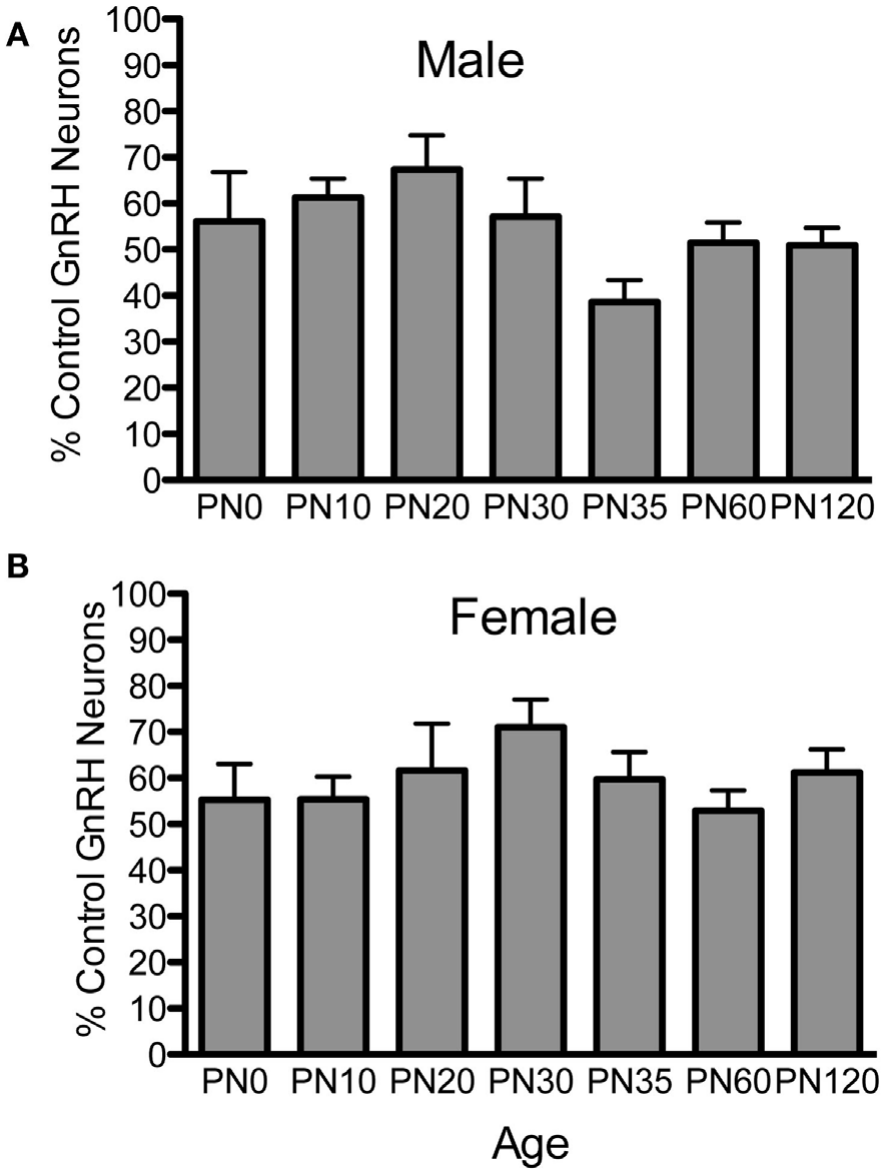
**Percent GnRH neurons in *Fgf8* Het male (A) and female (B) mice**. The number of GnRH neurons in each *Fgf8* Het mouse was normalized against the mean of GnRH neurons in the same age and sex group. Each bar = mean ± SEM; *N* = 5–6. No differences among age groups were observed in either male or female *Fgf8* Het mice.

To examine if postnatal loss of GnRH neurons occurred in *Fgf8* Het mice, GnRH neurons in PN0, 10, 20, 25, 30, 35, 60, and 120 male and female mice were analyzed (Figures 1 and 2). For males, two-way ANOVA revealed a significant effect of genotype [*F*(1, 67) = 152.5; *P* < 0.0001], age [*F*(6, 67) = 4.7; *P* = 0.0005], but no genotype × age interaction [*F*(6, 67) = 2.2; *P* = 0.053] on GnRH neuron number in males (Figure 1A). *Post hoc* test revealed a significant reduction of GnRH neurons in *Fgf8* Het males compared to WT males in every age group examined starting at PN0 (Figure 1A). When GnRH neuronal count in *Fgf8* Het males was analyzed as percent of GnRH neurons found in age-matched WT males (Figure 2A), Kruskal–Wallis test detected no significant differences across the ages examined (*P* = 0.14), suggesting *Fgf8* deficiency did not contribute to postnatal GnRH neurons loss within the first 120 days of birth. For females, two-way ANOVA revealed a significant effect of genotype [*F*(1, 68) = 119.4; *P* < 0.0001], age [*F*(6, 68) = 3.16; *P* < 0.0086], but no genotype × age interaction [*F*(6, 68) = 0.67; *P* = 0.67] on GnRH neuron number (Figure 1B). Similar to males, *post hoc* test also revealed a significant reduction of GnRH neurons in *Fgf8* Het females compared to WT females in every age group examined (Figure 1B). When GnRH neuronal count in *Fgf8* Het females was analyzed as percent of GnRH neurons found in age-matched WT females (Figure 2B), Kruskal–Wallis test, again, detected no significant differences across the ages examined (*P* = 0.47). Averaging all time points examined, male and female *Fgf8* Het mice had 55.3 ± 2.8 and 59.6 ± 2.5%, respectively, of the normal complement of GnRH neurons found in WT controls. Representative images of GnRH neurons near the OVLT in PN35 WT and *Fgf8* Het mice are shown (Figures 1C–F).

**Figure 3:**
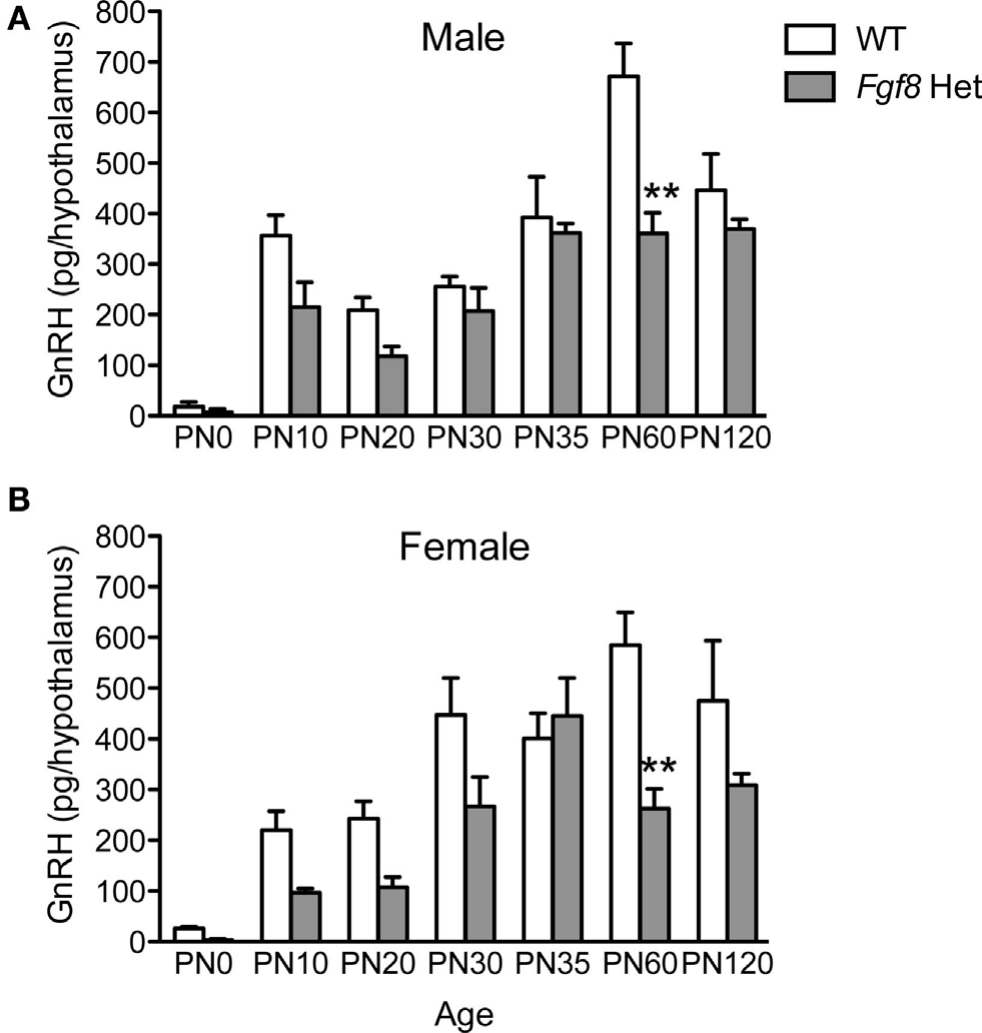
**Hypothalamic GnRH content in PN0–120 male (A) and female (B) WT and *Fgf8* Het mice**. In both sexes, GnRH contents were significantly reduced in *Fgf8* Het mice on PN60. Each bar = mean ± SEM; *N* = 3–5 for PN0 and 5–11 for other ages. **P < 0.01 compared to WT of the same age.

Next, we examined the levels of immunoassayable GnRH peptide in the hypothalami of WT and *Fgf8* Het mice at different ages and in both sexes. For males, two-way ANOVA revealed a significant effect of genotype [*F*(1, 86) = 14.93; *P* < 0.0002], age [*F*(6, 86) = 24.1; *P* < 0.0001], and genotype × age interaction [*F*(6, 86) = 2.91; *P* = 0.01] on hypothalamic GnRH (Figure 3A). *Post hoc* test revealed a significant reduction of hypothalamic GnRH in *Fgf8* Het males only on PN60 (Figure 3A). For females, two-way ANOVA revealed a significant effect of genotype [*F*(1, 91) = 18.45; *P* < 0.0001], age [*F*(6, 91) = 15.96; *P* < 0.0001], and genotype × age interaction [*F*(6, 91) = 2.48; *P* = 0.029] on hypothalamic GnRH (Figure 3B). Similar to males, *post hoc* test revealed a significant reduction of hypothalamic GnRH in *Fgf8* Het females only on PN60 (Figure 3B).

**Figure 4:**
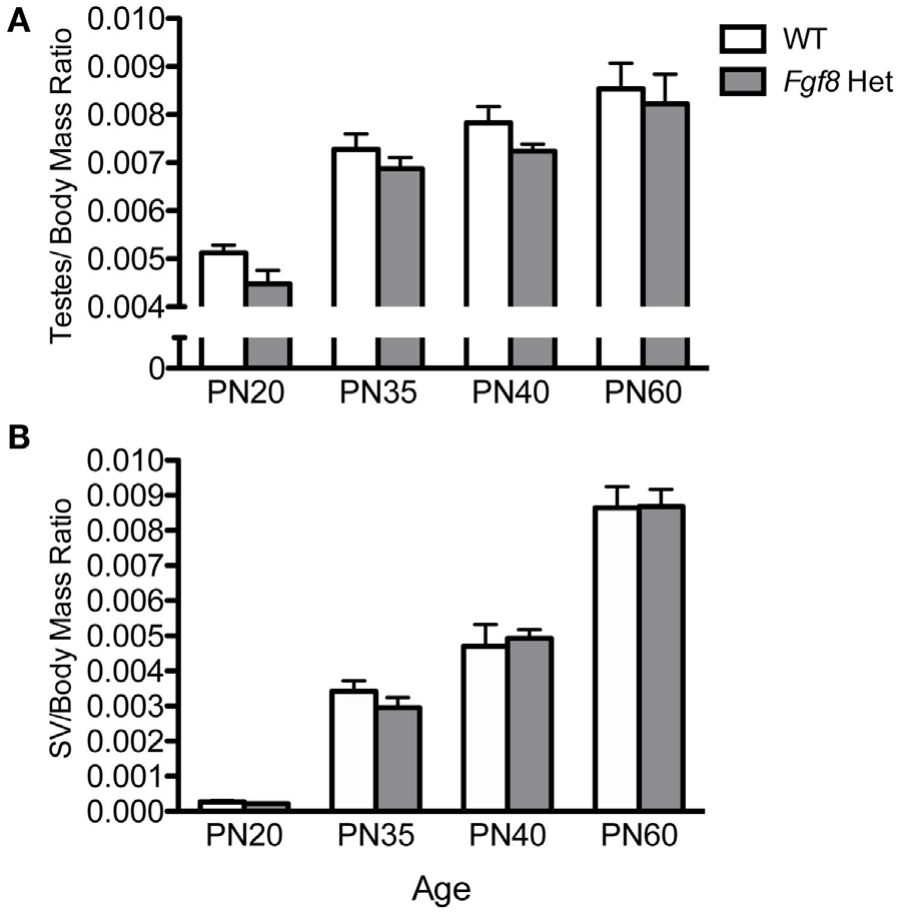
**Testes/body mass ratio (A) and SV/body mass ratio (B) in male PN20–60 WT and *Fgf8* Het mice**. Each bar = mean ± SEM; *N* = 5–11. No differences among age groups were observed in either parameter.

Female *Fgf8* Het mice were previously shown to exhibit delayed puberty and slightly disrupted estrous cycle (26); however, male *Fgf8* Het mice have not been examined for their reproductive parameters before, during, and after puberty. To address this, we initially compared testes/body mass and SV/body mass ratios of WT and *Fgf8* Het males at an age range that encompassed pre-pubertal (PN20), pubertal (PN35–40), and post-pubertal (PN60) periods. Two-way ANOVA revealed a significant effect of age [*F*(3, 46) = 35.1; *P* < 0.0001], but not genotype [*F*(1, 46) = 3.69; *P* < 0.061] or genotype × age interaction [*F*(3, 46) = 0.09; *P* = 0.96] on testes/body mass ratio (Figure 4A). Similarly, two-way ANOVA revealed a significant effect of age [*F*(3, 47) = 146.1; *P* < 0.0001], but not genotype [*F*(1, 47) = 0.06; *P* < 0.82] or genotype × age interaction [*F*(3, 47) = 3.52; *P* = 0.3] on SV/body mass ratio (Figure 4B). Overall, *Fgf8* deficiency had little effects on testicular and SV growth over the ages examined.

**Figure 5:**
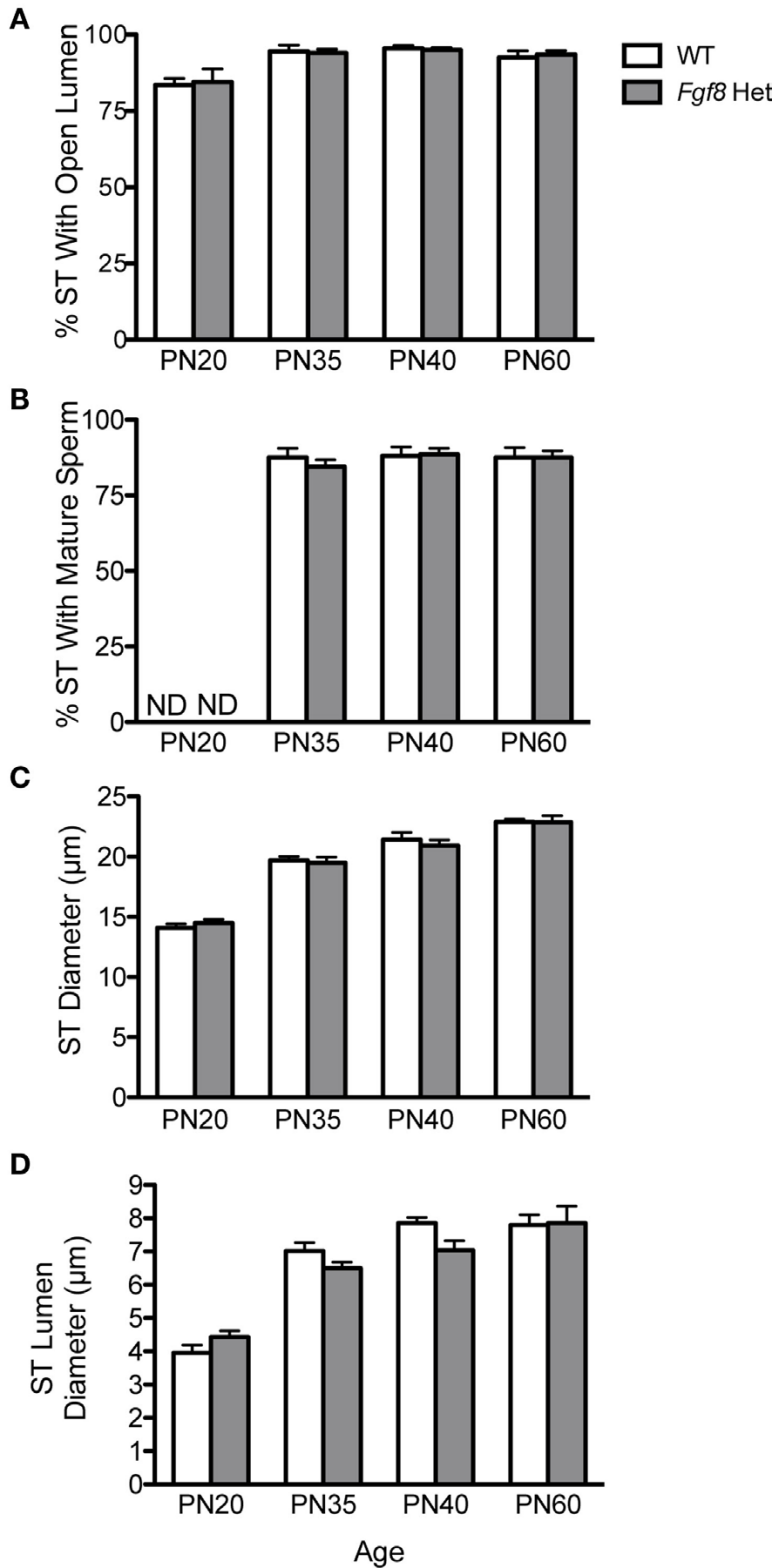
**Percent ST with open lumen (A), percent ST with mature sperm (B), ST diameter (C), and ST luminal diameter (D) in male PN20–60 WT and *Fgf8* Het mice**. Each bar = mean ± SEM; *N* = 5. ND = non-detectable. No differences among age groups were observed in any of the testicular parameters.

Testicular growth alone was not indicative of spermatogenic capacity, thus we performed morphometric analyses on several testicular histological parameters in WT and *Fgf8* Het males of different ages (Figure 5). Overall, two-way ANOVA revealed a significant effect of age on% ST with open lumen [*F*(3, 32) = 11.39; *P* < 0.0001] (Figure 5A), %ST with mature sperm [*F*(3, 32) = 702.3; *P* < 0.0001] (Figure 5B), ST diameter [*F*(3, 32) = 143.1; *P* < 0.0001] (Figure 5C), and ST luminal diameter [*F*(3, 32) = 66.6; *P* < 0.0001] (Figure 5D); however, no effects of genotype or genotype × age interaction were detected in any of these parameters, suggesting normal testicular function in *Fgf8* Het males over the ages examined (Figures 5A–D).

**Figure 6:**
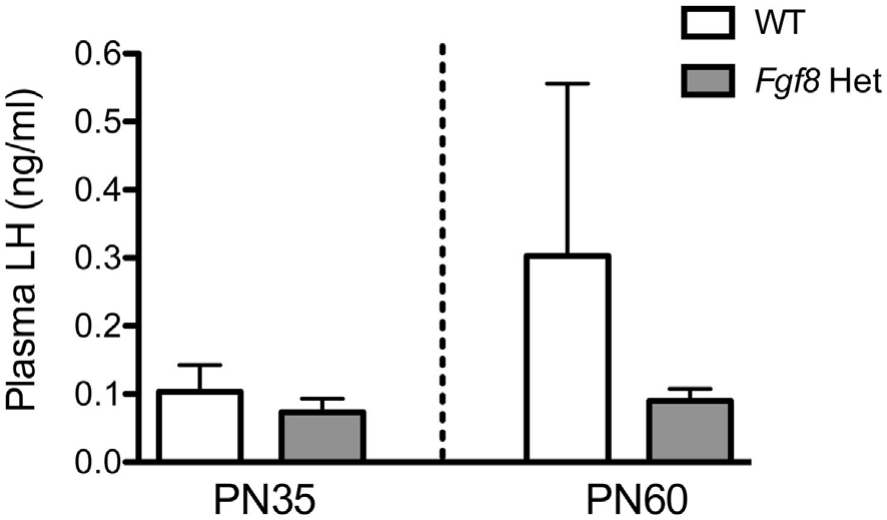
**Plasma LH in male PN35 and PN60 *Fgf8* Het mice**. Each bar = mean ± SEM; *N* = 6 for PN35 and *N* = 10–11 for PN60. No effects of genotype or age were observed.

Lastly, we measured plasma LH in PN35 and PN60 WT and *Fgf8* males. These are the two ages in which we observed no (PN35) and a significant (PN60) effect of genotype on hypothalamic GnRH content (Figure 3A). No significant effects of genotype, age, or age × genotype interaction on plasma LH were observed (Figure 6).

## Discussion

Three key findings emerge from the present study. First, *Fgf8* Het mice lost their GnRH neurons prenatally with no additional postnatal loss detected. Second, despite having an innately defective GnRH system, *Fgf8* Het mice had normal levels of the GnRH peptide through most pre-pubertal and pubertal periods examined and exhibited GnRH peptide deficiency only at a specific age (PN60) after puberty. Lastly, pubertal transition, gaged by the mass of testes and SV and testicular morphology, was largely normal in *Fgf8* Het males. Overall, our data underscored the inherent robustness of a neuroendocrine system that could compensate for the loss of almost half of its neuronal population with little reproductive consequence.

At present, the mechanism of prenatal GnRH neuronal loss in *Fgf8* Het mice is unclear. *Fgf8* Het mice had only 50% GnRH neurons at birth (20, 22) but were reported to have a normal complement of GnRH neurons on embryonic day (E) 15.5 (22). The most parsimonious explanation for this discrepancy was that *Fgf8* Het mice lost 50% of their GnRH neurons between E15.5 and PN0 due to the failure of these neurons to migrate, survive, or remain differentiated. We considered migratory defect unlikely since most GnRH neurons in *Fgf8* Het embryos were reported to reach the forebrain by E15.5 (22), an age in which the majority of neurons have completed their migration (29, 30). Of further interest, the loss of GnRH neurons observed in newborn *Fgf8* Het mice persisted in all later ages examined, suggesting this loss was irreversible for the first four postnatal months and possibly the rest of the animals’ lives. Moreover, no sex differences existed in the impact of *Fgf8* deficiency on the GnRH neuronal population. These observations collectively supported the notion that the loss of GnRH neurons in *Fgf8* Het mice was an irreversible developmental event that likely occurred after these neurons transitioned from the nose into the forebrain environment. Whether Fgf8 is needed to support the survival or differentiation of the developing GnRH neurons requires further interrogation, preferably using lineage-tracing techniques to permanently mark these neurons.

Although male and female *Fgf8* Het mice exhibited consistent GnRH neuronal loss at all postnatal time points examined, a significant reduction in hypothalamic GnRH peptide levels was observed only on PN60 of both sexes (Figure 3). This observation carried several interesting implications, the foremost being that a defective GnRH system could largely compensate for the developmental GnRH deficit. The murine GnRH system was reported to be highly redundant, and a GnRH neuronal population that had been reduced by 90% could continue to sustain normal male reproduction (31). Our results suggested that *Fgf8* Het mice may increase the level of bioavailable GnRH peptide via several mechanisms (see paragraph below) despite reduced GnRH neuron numbers. That said, *Fgf8* Het females experienced delayed VO and first estrus (26), suggesting the compensation may not always be complete. Interestingly, Herbison et al. (31) also reported that the consequence of reduced GnRH neurons may be more deleterious in females than in males. At present, it is unclear why only PN60 *Fgf8* Het mice displayed a reduction in immunoassayable hypothalamic GnRH. Postnatally, hypothalamic GnRH peptide levels in mice peaked between PN30 and 40 (32) and *GnRH* transcript levels peaked between PN40 and 60 (33). This time course suggested that the GnRH system may have a robust synthetic machinery before and around puberty, but the synthetic drive plateaued after PN40. We hypothesize that *Fgf8* Het mice may experience an earlier plateau than WT mice, leading to the observed pattern.

The mechanism leading to normal GnRH peptide levels in *Fgf8* Het mice of most ages is unclear at present. The fewer GnRH neurons in *Fgf8* Het mice could compensate by increasing mRNA levels via enhanced transcription and mRNA stability or by promoting translation efficiency and post-translational modification (34, 35). These mechanisms contribute to the accumulation of GnRH peptide and should be explored in the future. It is also interesting to note that the anteroventral periventricular (AVPV) kisspeptin system of *Fgf8* Het females was downregulated on PN30 but normal at other pre- and post-pubertal periods (26). These results suggested that *Fgf8* deficiency, besides reducing GnRH neurons, also reduced AVPV kisspeptin neurons age-specifically to delay female puberty. However, neither the AVPV nor the arcuate kisspeptin system in *Fgf8* Het males had been examined, and their roles in safeguarding normal pubertal transition of these mice remain unclear.

Our analysis of testicular and SV/body mass ratios revealed no differences in the growth of these two reproductive organs between WT and *Fgf8* Het mice. Since SV growth was androgen-dependent (36), these results suggested testicular steroidogenesis was not significantly altered in the mutant mice. Further analyses of testicular parameters such as %ST with open lumen and mature sperm as well as ST total and luminal diameters also revealed no genotype differences before, during, and after pubertal transition (Figure 5). Consistent with these observations, the fertility of these mice was thought to be normal (25).

Previous studies reported the elimination of the entire GnRH neuronal population in *Fgf8* homozygous hypomorphic mice (20, 22) despite the remaining presence of ~50% functional *Fgf8* transcript, suggesting the exquisite sensitivity of the murine GnRH system to Fgf8. Despite the importance of Fgf8 in this system, *Fgf8* Het mice have been consistently fertile in our colony. The lack of obvious male reproductive phenotype in *Fgf8* Het mice may initially appear to contradict the reported subfertility and HH in some patients with *Fgf8* haploinsufficiency (15, 24), but several explanations may account for this discrepancy. First, the reproductive phenotype of humans with *Fgf8* mutations has been difficult to predict due to variable penetrance and, in some cases, may be an oligogenic condition requiring additional disease genes to become fully manifested (15, 24, 37). In other words, some HH patients with *Fgf8* haploinsufficiency may harbor additional mutations yet to be detected. Second, earlier studies transplanting GnRH neurons into hypogonadal (*hpg*) mice (38) showed the successful transplantation of as few as 1–3 GnRH neurons could restore several reproductive parameters (39–41), suggesting the murine GnRH system is functionally robust and that 200–300 GnRH neurons in *Fgf8* Het mice should adequately support reproduction. Third, we have not yet fully assessed the fertility of male *Fgf8* Het mice, and such assessment may reveal more subtle defects. Indeed, although male *hpg* mice transplanted with a small number of GnRH neurons exhibited elevated ­gonadotropin levels and underwent testicular and SV growth (42, 43), they did not exhibit normal gonadotropin and androgen levels and failed to ejaculate and impregnate females (42, 43). It would be interesting to assess if *Fgf8* Het males exhibited normal mating frequency and fecundity. Lastly, the functional capacity of murine GnRH neurons may be highly dependent on the environmental condition. HH or suboptimal gonadal function stemming from reduced GnRH neurons could be reversed in mice simply by interactions with the opposite sex (44, 45), implicating high levels of functional plasticity. Future studies should assess the fertility of *Fgf8* Het mice under suboptimal environmental conditions, such as caloric restriction or psychosocial stress, to interrogate the functional outcome of a reduced GnRH system when challenged.

In conclusion, the present study reinforces the idea that a developmentally compromised GnRH system remains highly plastic. Except on PN60, the GnRH system in *Fgf8* hypomorphic mice of various ages may compensate for the reduced GnRH neuron numbers by increasing the levels of bioavailable GnRH. The significant reduction in GnRH neuron number had no ­discernible impact on male testicular parameters or circulating LH as they transitioned through puberty. Overall, the present study showed that in spite of a significantly reduced GnRH system, compensatory mechanisms exist to drive sexual maturation and support adult gonadal function.

## Conflict of Interest Statement

The authors declare that the research was conducted in the absence of any commercial or financial relationships that could be construed as a potential conflict of interest.
